# Genome-Wide Association Study Based on Random Regression Model Reveals Candidate Genes Associated with Longitudinal Data in Chinese Simmental Beef Cattle

**DOI:** 10.3390/ani11092524

**Published:** 2021-08-27

**Authors:** Lili Du, Xinghai Duan, Bingxing An, Tianpeng Chang, Mang Liang, Lingyang Xu, Lupei Zhang, Junya Li, Guangxin E, Huijiang Gao

**Affiliations:** 1 Institute of Animal Science, Chinese Academy of Agricultural Sciences, Beijing 100193, China; 82101192341@caas.cn (L.D.); 82101192337@caas.cn (X.D.); 82101191203@caas.cn (B.A.); 82101181188@caas.cn (T.C.); 82101182356@caas.cn (M.L.); xulingyang@caas.cn (L.X.); zhanglupei@caas.cn (L.Z.); lijunya@caas.cn (J.L.); 2 College of Animal Science and Technology, Southwest University, Chongqing 400715, China

**Keywords:** random regression model, longitudinal trait, GWAS, Chinese Simmental beef cattle

## Abstract

**Simple Summary:**

Genome-wide association study (GWAS) has become the main approach for detecting functional genes that affects complex traits. For growth traits, the conventional GWAS method can only deal with the single-record traits observed at specific time points, rather than the longitudinal traits measured at multiple time points. Previous studies have reported the random regression model (RRM) for longitudinal data could overcome the limitation of the traditional GWAS model. Here, we present an association analysis based on RRM (GWAS-RRM) for 808 Chinese Simmental beef cattle at four stages of age. Ultimately, 37 significant single-nucleotide polymorphisms (SNPs) and several important candidate genes were screened to be associated with the body weight. Enrichment analysis showed these genes were significantly enriched in the signaling transduction pathway and lipid metabolism. This study not only offers a further understanding of the genetic basis for growth traits in beef cattle, but also provides a robust analytics tool for longitudinal traits in various species.

**Abstract:**

Body weight (BW) is an important longitudinal trait that directly described the growth gain of bovine in production. However, previous genome-wide association study (GWAS) mainly focused on the single-record traits, with less attention paid to longitudinal traits. Compared with traditional GWAS models, the association studies based on the random regression model (GWAS-RRM) have better performance in the control of the false positive rate through considering time-stage effects. In this study, the BW trait data were collected from 808 Chinese Simmental beef cattle aged 0, 6, 12, and 18 months, then we performed a GWAS-RRM to fit the time-varied SNP effect. The results showed a total of 37 significant SNPs were associated with BW. Gene functional annotation and enrichment analysis indicated *FGF4*, *ANGPT4*, *PLA2G4A*, and *ITGA5* were promising candidate genes for BW. Moreover, these genes were significantly enriched in the signaling transduction pathway and lipid metabolism. These findings will provide prior molecular information for bovine gene-based selection, as well as facilitate the extensive application of GWAS-RRM in domestic animals.

## 1. Introduction

With the development of the high-throughput chip technologies and the completion of whole-genome sequencing of swine [1], cattle [2], sheep [3], chicken [4], and other domestic animals [5], genome-wide association study (GWAS) has become an indispensable statistical method that can detect significant single-nucleotide polymorphisms (SNPs) and functional genes affecting economical traits in domestic animals, including growth traits, fertility traits [6], and meat quality [7], which greatly contributes to improving animal breeding and reproduction.

Numerous GWAS have been widely performed on single-record traits of beef cattle, such as birth weight, weaning weight, and yearling weight (YW) [8,9], and several significant SNPs and candidate genes were mapped. Buzanskas et al. revealed four SNPs significantly associated with the BW trait in Canchim beef cattle [10]. Zhuang et al. performed the weighted single-step GWAS in 744 Chinese Simmental beef cattle with 770K BovineHD SNP BeadChip, concluding TBC1D5 and MYH10 were associated with birth weight at the age of 18 months and YW [11], respectively, of which MYH10 was also identified to be related to chicken growth traits [12]. Amounts of candidate genes and significant SNPs relevant to the BW trait have been identified and submitted to Cattle Quantitative Trait Locus Database (Cattle QTLdb) [13]. Longitudinal traits are defined as a type of functional traits that are observed repeatedly over multiple time points during an organism’s life cycle [14], such as test-day milk yield and body condition scores in dairy cattle, periodic body weight, and daily gain in beef cattle, litter size in swine, and egg production in chicken, etc. Compared with single-record traits, the longitudinal traits classified into multi-record traits could better reflect the growth and development pattern of livestock with time. At present, there are three main models for GWAS analysis of longitudinal traits, namely the two-stage analysis method [15], the point-by-point analysis method, and the analysis method based on the random regression model (RRM) [16]. Among these analysis ideas, GWAS analysis based on RRM (GWAS-RRM) could result in the high accuracy of estimated breeding values and the decrease of false positive rate (FPR) in animals breeding [17]. Consistent with these findings, simulation studies conducted by Ning et al. showed GWAS-RRM for longitudinal traits could decrease FPR and increase statistical powers in the detection of quantitative trait nucleotide (QTN) [18], which enabled large-scale GWAS analysis for longitudinal traits [14]. Emamgholi et al. also proposed GWAS-RRM analysis could improve the selection accuracy for the trajectory of feed intake traits in the F2 chickens’ population [19]. Additionally, Oliveira et al. performed GWAS-RRM to detect candidate genes associated with milk production traits (milk, fat, and protein yields) in three breeds of dairy cattle. They found there were differential expression patterns of candidate genes underlying the phenotypic expression across breeds and lactation stages [20]. The same method was conducted on Duroc for daily feed intake and average daily weight, and results showed candidate genes associated with these traits were mainly involved in metabolite homeostasis and insulin signaling [21]. Taken together, these studies indicated that GWAS-RRM has been widely applied in the genetic evaluation of longitudinal traits in dairy cattle, especially for its milk production, but not in beef cattle [22,23].

Body weight (BW) is an economically important longitudinal trait in beef cattle that greatly influences growth performance [10]. Until now, longitudinal traits have been increasingly available in GWAS-RRM for the identification of significant SNPs and promising candidate genes that influence economically important traits in livestock over time; however, less attention was given to longitudinal traits in beef cattle. In the present study, GWAS-RRM was performed on the BW trait of 808 Chinese Simmental beef cattle at the age of 0, 6, 12, and 18 months to identify important significant SNPs and promising candidate genes associated with this trait. These findings will contribute to understanding the molecular basis of growth and development traits in beef cattle, and provide insights into the studies of longitudinal traits in other domestic animals.

## 2. Materials and Methods

### 2.1. Animal Resource and Phenotypes Recording

All animals and protocols in the study were approved by the ethics committee of the Institute of Animal Sciences, Chinese Academy of Agricultural Sciences (CAAS), Beijing, China (approval number: RNL09/07). A total of 808 male individuals in this study were derived from the Chinese Simmental beef cattle resource population established in Ulgai, Xilingol League, Inner Mongolia of China from 2008 to 2014. After weaning, the cattle were moved to the Beijing Jinweifuren fattening farm for fattening in the same feeding strategies and management conditions. Body weight was measured for each individual at 0, 6, 12, and 18 months after birth, respectively. Here, the body weight data were consistent with the data in the study by Duan et al. [24].

### 2.2. Genotyping and Quality Control

Blood samples for the experimental population were collected along with the periodic quarantine inspection of the farm. Genomic DNA was isolated from blood samples using the TIANamp Blood DNA Kit (Tiangen Biotech Co.Ltd., Beijing, China), and the high-quality DNAs with the A260/280 ratio ranging 1.8–2.0 were considered for further analysis. In this study, the Illumina BovineHD Beadchip with 774,660 SNPs (Illumina Inc., San Diego, CA, USA) was used for qualified DNAs genotyping and Illumina’s Infinium II Assay was selected as the genotyping platform. The SNPs were uniformly distributed on the whole bovine genome with a mean inter-marker space of 3.43 kb. SNP chips were scanned and analyzed using the Infinium GenomeStudio software (Illumina Inc., San Diego, CA, USA). PLINK v1.9 (http://zzz.bwh.harvard.edu/plink/, accessed on 1 July 2021) was used for quality control of SNPs according to the following empirical excluded criteria: (1) minor allele frequency (MAF) < 0.01; (2) SNP call rate (CR) < 95%; (3) Hardy–Weinberg equilibrium value *p* < 1 × 10^−6^; (4) Mendelian error of SNP genotype above 2%; (5) Individuals with more than 10% SNPs deletion; (6) SNP marker sites with missing chromosomal location information. All the misplaced and duplicated SNPs were also excluded from the analysis. Ultimately, 671,192 SNPs with an average marker interval of 3 kb on 29 autosomal chromosomes remained for subsequent analysis.

### 2.3. Population Stratification

Population stratification usually caused serious FPR in GWAS analysis. Here, we performed a principal component analysis (PCA) by PLINK v1.9 [25]. Our previous work demonstrated the first two principal components had been selected as covariances to eliminate the influence of population stratification [24].

### 2.4. Genome-Wide Association Study Based on the Random Regression Model

The general expression of the random regression model is as follows:

(1) $$y_{tijk}=F_{i}+f{\left(t\right)}_{j}+r{\left(a,x,m_{1}\right)}_{k}+r{\left(p,x,m_{2}\right)}_{k}+e_{ijkt}$$

where $\;y_{tijk}$ is the measured value of individual *k* at time *t*; $F_{i}$ is the time-independent fixed environmental effect; $f{\left(t\right)}_{j}$ is fixed regression function, reflecting the average change trend of phenotypic values of animals in group *j* with time *t*; $r{\left(a,x,m_{1}\right)}_{k}$ and $r{\left(p,x,m_{2}\right)}_{k}$ are random regression functions, which represent time-varied additive genetic effect and permanent environmental effect for individual *k*, respectively; a and p are the random regression coefficients of additive genetic effect and permanent environmental effect, respectively; $m_{1}$ and $m_{2}$ are the orders of the corresponding regression function; x is a covariable; $e_{ijkt}$ is the time-independent random residual for each measurement of individual *k* at time *t*. Here, $f{\left(t\right)}_{j}$, $r{\left(a,x,m_{1}\right)}_{k}$ and $r{\left(p,x,m_{2}\right)}_{k}$ can be described as the Legendre polynomial regression for a set of basis functions, specific form as follows:

(2) $$f{\left(t\right)}_{j}=\overset{m_{f}}{\underset{l=0}{\sum }}b_{jl}\phi_{tjkl},\;r{\left(a,x,m_{1}\right)}_{k}=\overset{m_{r1}}{\underset{l=0}{\sum }}a_{jkl}\phi_{tjkl},\;r{\left(p,x,m_{2}\right)}_{k}=\overset{m_{r2}}{\underset{l=0}{\sum }}p_{jkl}\phi_{tjkl}$$

where $b_{jl}$ is the *l*th fixed regression coefficient; $a_{jkl}$ and $p_{jkl}$ are the *l*th random regression coefficients for additive genetic effect and permanent environmental effect of the *k*th individual, respectively; $m_{f}$, $m_{r1}$, and $m_{r2}$ are the orders of corresponding basis functions; The orders of different basis functions can be determined by model selection criteria proposed by Das et al. [26]. For instance, in the present study, Akaike information criterion (AIC) and Bayesian Information Criterion (BIC) were used to determine a fifth-order basis function for the population mean, a third order for additive genetic effects and a fifth order for permanent environmental effects were best fit to the data for BW trait. $\phi_{tjkl}$. represents the value of the *l*th basis function at time t, i.e., the value of the Legendre polynomial (covariable).

The Equation (1) can be denoted as:

(3) $$y=X_{1}f+X_{2}b+Z_{1}a+Z_{2}p+e$$

We assume that there are *m* out of *n* individuals for which phenotypic values are measured; *m_k_* represents the phenotypic record for individual *k* and the total number of records for all individuals is $m=\sum_{k=1}^{n}m_{k}$. Therefore, y is the vector (*m* × 1) of phenotypic values of all individuals. The parameters in the equation are defined as follows: Parameter **f** is a vector of fixed environmental effect and parameter $X_{1}$ is the corresponding incidence matrix; Parameter b is the vector (*m_f_* + 1) of fixed regression coefficients; Parameter a ($n\times \left(m_{r}+1\right)$) and p ($m\times \left(m_{r}+1\right)\;$) are the vector of random regression coefficients for additive genetic effect and permanent environmental effect respectively for each individual; Parameter $X_{2}$, $Z_{1}$, and $Z_{2}$ are the corresponding covariance matrix; e is the vector of random residuals.

For matrix form (3), we have the (co) variance matrices of all random effects:

(4) $$Var\left[\begin{array}{c} a\\ p \end{array}\right]=G=\left[\begin{array}{cc} A⊗D & 0\\ 0 & I⊗P \end{array}\right]\;\mathrm{and}\;Var\left(e\right)=R=I\sigma_{e}^{2}$$

here, A is the numerator relationship matrix based on pedigree information; D and P are the variance-covariance matrix of random regression coefficients for additive genetic effect and permanent environmental effect, respectively; I is the identity matrix; $\sigma_{e}^{2}$ is the residual variance; The symbol “⊗” represents the Kronecker product. Therefore, the mixed model equations can be represented as:

(5) $$\left[\begin{array}{cccc} X_{1}^{\prime }X_{1} & X_{1}^{\prime }X_{2} & X^{\prime }Z_{1} & X^{\prime }Z_{2}\\ X_{2}^{\prime }X_{1} & X_{2}^{\prime }X_{2} & X_{2}^{\prime }Z_{1} & X_{2}^{\prime }Z_{2}\\ Z_{1}^{\prime }X_{1} & Z_{1}^{\prime }X_{2} & Z_{1}^{\prime }Z_{1}+\sigma_{e}^{2}A^{-1}⊗D^{-1} & Z_{1}^{\prime }Z_{2}\\ Z_{2}^{\prime }X_{1} & Z_{2}^{\prime }X_{2} & Z_{2}^{\prime }Z_{1} & Z_{2}^{\prime }Z_{2}+\sigma_{e}^{2}I⊗P^{-1} \end{array}\right]\left[\begin{array}{c} \hat{f}\\ \hat{b}\\ \hat{a}\\ \hat{p} \end{array}\right]=\left[\begin{array}{c} X_{1}^{\prime }y\\ X_{2}^{\prime }y\\ Z_{1}^{\prime }y\\ Z_{2}^{\prime }y \end{array}\right]\;\mathrm{Note}:\;G^{-1}={\left[\begin{array}{cc} A⊗D & 0\\ 0 & I⊗P \end{array}\right]}^{-1}=\left[\begin{array}{cc} A^{-1}⊗D^{-1} & 0\\ 0 & I⊗P^{-1} \end{array}\right]$$

Based on the general random regression model, the functional GWAS model (fGWAS-C) for the association analysis of longitudinal traits has been proposed by Ning, C. [18], which adds an additional fixed regression term to Equation (1) to account for the effect of the SNP. Th fGWAS-C can be expressed as:

(6) $$y_{tijk}=f{\left(t\right)}_{j}+x_{i}SNP\left(t\right)+r{\left(a,x,m_{1}\right)}_{k}+r{\left(p,x,m_{2}\right)}_{k}+e_{ijkt}$$

here, $x_{i}$ is a genotype indicator variable that is coded as 0, 1, or 2 for the three genotypes, aa, Aa, and AA, respectively. SNP(t) represents the time-varied additive effect for each SNP at time *t*. The function expression of SNP(t) is:

(7) $$SNP\left(t\right)=\overset{m_{f}}{\underset{l=0}{\sum }}\eta_{l}\phi_{tjkl}$$

where $m_{f}$ is the order of basis functions for the time-varied SNP effect; $\eta_{l}$ is the *l*th fixed regression coefficient of SNP additive effect; $\phi_{tjkl}$ is the value of the *l*th basis function at time t.

The threshold *p*-value for GWAS analysis was calculated as follows:

*p* = FDR × n/m (8)

where FDR was usually set at 0.05; n is the number of SNPs with *p* < 0.05; m is the total number of SNPs after quality control [27].

### 2.5. Detection and Functional Enrichment of Candidate Gene

The significant SNPs associated with BW were screened according to the threshold *p*-value and then the Ensembl–BioMart was used to match these SNPs with the bovine reference genome UMD 3.1 (http://www.ensembl.org/Biomart, accessed on 1 July 2021). Candidate genes in the target region were screened through the National Center for Biotechnology Information (NCBI) database (https://www.ncbi.nlm.nih.gov/, accessed on 1 July 2021), cattle QTLdb [13], and previous relevant studies. Then Gene Ontology (GO) and Kyoto Encyclopedia of Genes and Genomes (KEGG) enrichment analysis revealed the function of candidate genes, and protein-protein interaction (PPI) network explored the interaction between node proteins encoded by these genes [28]. The *p*-value adjusted using the Benjamini–Hochberg approach (*p*-value < 0.05) was considered to be the threshold value for significantly enriched GO terms and pathways. Using ToppCluster and Cytoscape v3.8.0 to plot the network diagram between candidate genes and their belonged GO terms/pathways. The STRING (https://string-db.org/, accessed on 1 July 2021) was used to perform PPI network analysis.

### 2.6. Statistical Analysis

Using SPSS v20.0 to calculate the measurement values of body weight at four growth stages. All data were expressed as means ± standard deviation (M ± SD). Microsoft Excel 2010 software was used to check up the data with normality test.

## 3. Results

### 3.1. Data Statistics of Body Weight

**Figure 1:**
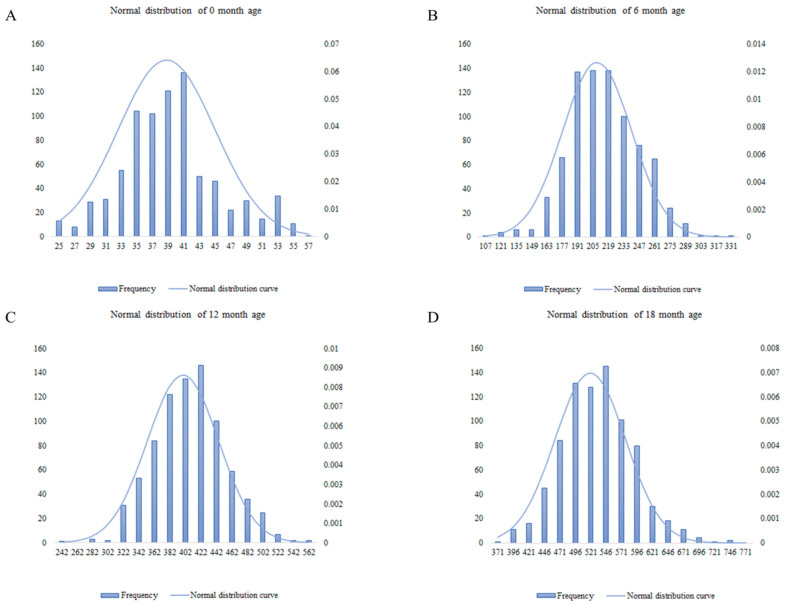
Normal distribution of bovine body weight of the test population at different months of age. A shows the normal distribution of body weight at 0 months of age; B shows the normal distribution of body weight at 6 months of age; C shows the normal distribution of body weight at 12 months of age; D shows the normal distribution of body weight at 18 months of age. In the figure, the abscissa represents weight, the left ordinate represents frequency, and the right ordinate represents normal function values.

The BW values of Chinese Simmental beef cattle at 0, 6, 12, and 18 months of age were tested for normality. As shown in Figure 1, the phenotypic values of the individuals at different months of age presented normal distributions, which was in line with the hypothesis of the model and could be used for subsequent association analysis. The descriptive statistics of the BW trait at different months of age were presented in Supplementary Table S1.

### 3.2. Genome-Wide Association Study Based on the Random Regression Model

**Figure 2:**
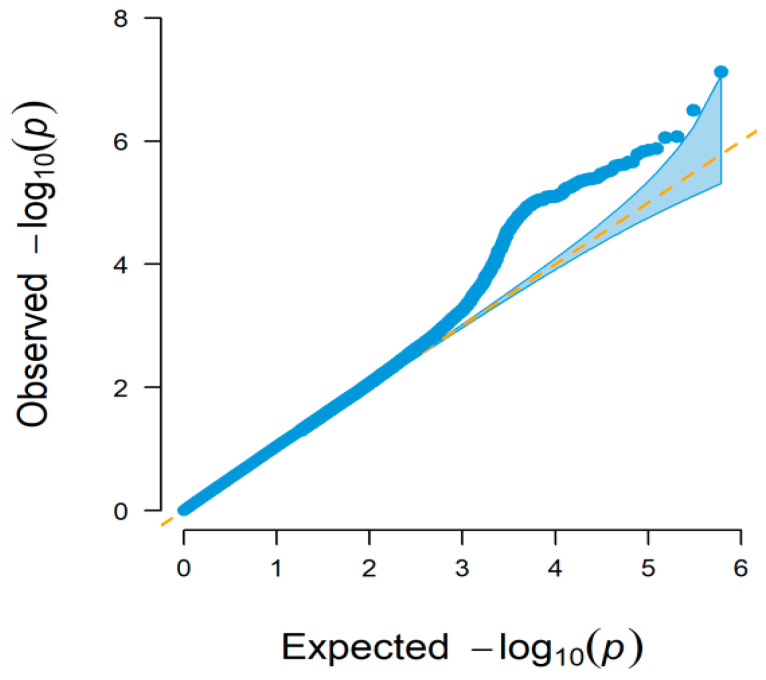
Quantile-quantile (Q-Q) plot of GWAS based on the random regression model. The x-axis and y-axis represent −log_10_ transformed expected *p*-values and observed *p*-values, respectively. The dots indicate −log_10_(*p*) of the SNPs and the diagonal line represents the expected values under the null hypothesis for no association.

**Figure 3:**
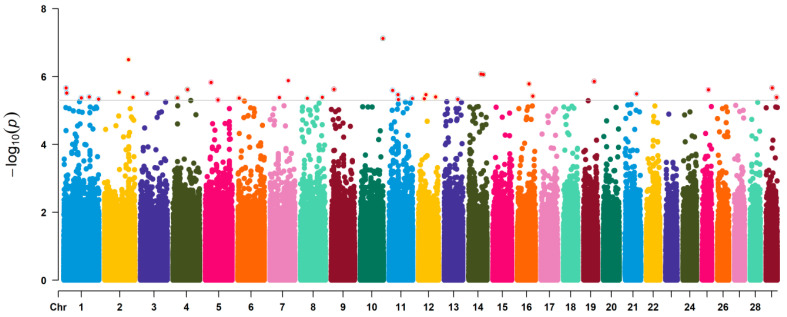
Manhattan plot of GWAS based on the random regression model. The horizontal axis shows the chromosomal position of the SNPs associated with body weight. The vertical axis indicates the absolute value of the −log_10_ (*p*) of individual SNPs.

The quantile-quantile (Q–Q) and Manhattan plots of GWAS-RRM analysis are shown in Figure 2 and Figure 3, respectively. Distributions of the observed −log_10_(*p*) versus expected −log_10_(*p*) in the Q–Q plot represented most points revolving around the 45° line, indicating there was no inflation or systematic bias in this study, as well as population stratification was well controlled. The Manhattan plots showed that a total of 37 significant SNPs associated with BW trait were identified, most of which were located on *Bos taurus* autosome (BAT) 1 (five SNPs), BAT 2 (three SNPs), BAT 11 (four SNPs), and BAT 12 (three SNPs). Two SNPs were found on each of the seven chromosomes (BAT 4, 5, 7, 8, 14, 16, and 29), and only one SNP was distributed on each of the eight chromosomes (BAT 3, 6, 9, 10, 13, 19, 21, and 25). The SNP with the smallest *p*-value (*p* = 7.55 × 10^−8^) was located on BAT 10: 101,577,026 bp (BovineHD1000029459). However, some SNPs had the lowest significance levels, i.e., the *p*-value of BovineHD1100004962 and BovineHD1100011885 located on BAT 11 was 2.54 × 10^−6^ and 3.42 × 10^−6^, respectively. The detailed information of all significant SNPs was displayed in Table 1 and Supplementary Table S2. 

### 3.3. Genes Detection

In this study, 37 significant SNPs were identified for BW trait, of which 33 were located within or near candidate genes through searching Ensembl, NCBI, and QTL databases. As shown in Table 1, significant SNPs on BAT5, BAT13, and BAT29 were within or close to genes *ITGA5*, *ANGPTL4*, and *FGF4*, respectively. Three significant SNPs were identified at the region of 26.11-33.58 Mb on BAT12, of which BvineHD1200027798 and BovineHD1200026844 were close to genes *SHISA2* and *CHMP3*, respectively. No candidate genes were detected in four significant SNPs including BovineHD0200018897, BovineHD0700012290, BovineHD1100030552, and BovineHD2900008350, located on BAT2, BAT7, BAT11, BAT12, and BAT29, respectively. The detailed information on these genes was listed in Supplementary Table S2.

### 3.4. Functional Annotation of Candidate Genes

**Figure 4:**
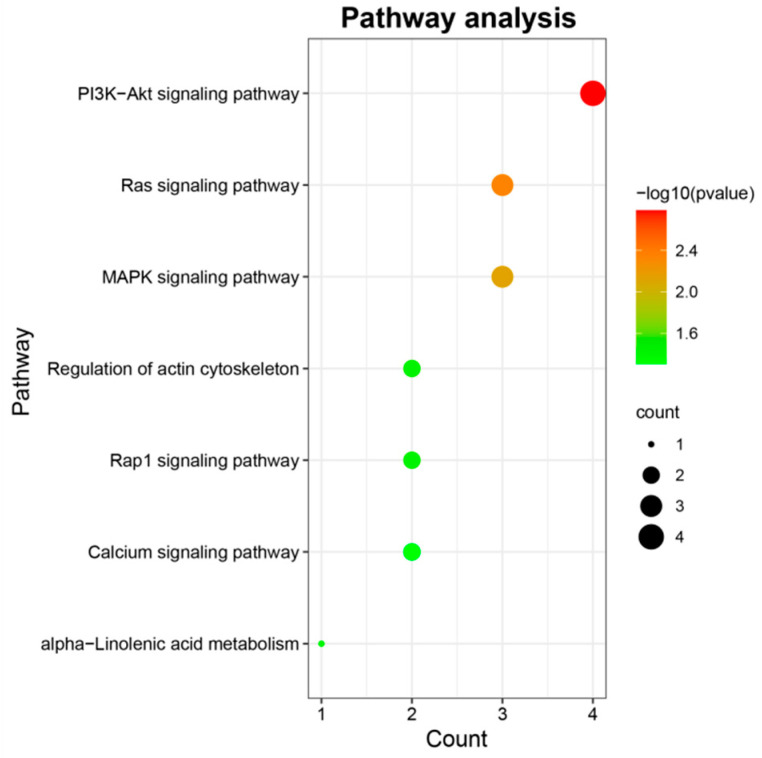
KEGG enrichment of detected genes. The x-axis and y-axis indicate the number of genes enriched per KEGG pathway and the most significantly enriched pathways, respectively. The greater the number of genes enriched in each pathway, the larger the corresponding bubbles.

**Figure 5:**
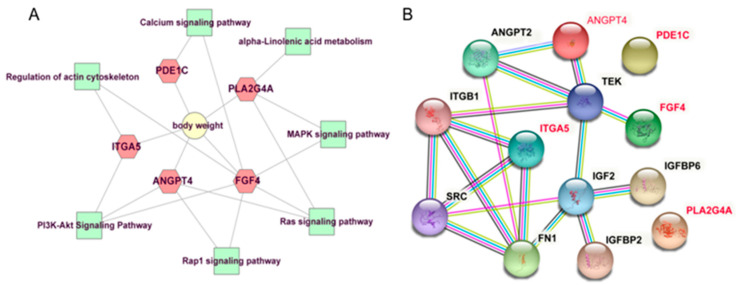
A represents the network diagram of the candidate genes affecting BW and their belonged pathways; B represents the PPI network of the candidate genes affecting BW. The genes marked in red represent potential candidate genes influencing BW in this study.

To further understand the function of candidate genes, GO and KEGG enrichment was performed using the R package “clusterProfiler” [29]. As shown in Figure 4, seven pathways were significantly enriched, of which five pathways were implicated in signal transduction, including PI3K-Akt signaling pathway (bta04151), Ras signaling pathway (bta04014), MAPK signaling pathway (bta04010), Rap1 signaling pathway (bta04015), and Calcium signaling pathway (bta04020); one pathway, regulation of actin cytoskeleton (bta04810), was involved in cell motility; one pathway was associated with lipid metabolism, namely alpha-Linolenic acid metabolism (bta00592). The information about these significant pathways is shown in Supplementary Table S3. No GO term was significantly enriched. Notably, *FGF4, ANGPT4*, *ITGA5*, and *PLA2G4A* involved in no less than two KEGG pathways deserved further attention and discussion. Additionally, based on animal QTLdb, the previous reports and gene function analysis, several candidate genes including *TBC1D5*, *SHISA2*, *PDE1C,* and *COCX6* were also identified to affect the BW trait. The information of these candidate genes was listed in Table 2. Numerous candidate genes known to be related to BW in domestic animals were presented in Supplementary Table S4. PPI network shown in Figure 5 visualized the interaction between node proteins encoded by corresponding candidate genes.

## 4. Discussion

Longitudinal traits can better reflect the growth and development patterns of livestock and poultry. Therefore, mapping and analyzing the significant SNPs and functional genes affecting longitudinal traits have important economic value for beef cattle breeding. It is worth noting that GWAS-RRM is the main approach to analysis longitudinal traits, which could better control FPR and improve the accuracy of estimated breeding values (EBVs) [17,18], thus improving the efficiency of GWAS analysis [30]. Body weight is an important longitudinal trait that greatly reflects bovine growth performance. Previous studies have shown skeletal muscle is involved in the structure and metabolic regulation of the body and its mass accounts for 40% of total body weight in animals [31], indicating the growth and development of animals are inseparable from muscle development. The actin cytoskeleton is an important muscle structure that regulates cell adhesion, cell proliferation, cell motility, and muscle contraction via the signals transduction from the extracellular matrix to the nucleus [32]. Ito et al. elucidated that the MAPK signaling pathway could stimulate the growth of skeletal muscle and cell proliferation [33]. Therefore, it could be concluded that the pathway of regulation of actin cytoskeleton and the MAPK signaling pathway might be potential candidate pathways affecting the BW trait via affecting the development of skeletal muscle. In the present study, several candidate genes (*FGF4*, *ANGPTL4*, *PLA2G4A*, and *ITGA5*) were significantly enriched in the above pathways, which implied these genes might play special roles in regulating the BW trait.

*Fibroblast growth factors* (*FGFs*) are important growth factors that participate in many developmental and physiological processes [34]. FGF signaling could be disrupted due to the mutations of *FGFs*, thus causing developmental disorders, i.e., skeletal diseases, infertility, and cancer [35]. Relevant studies have reported some members of the *FGF* family, *FGF4*, and *FGF14*, regulate fibroblasts formation and the development of growth traits such as BW trait [36,37]. The complete nucleotide sequence of the bovine *FGF4* was identified in three cattle breeds (panese Black, Japanese Shorthorn, and Holstein cattle) [38], and its coding exons encode 206 amino acid residues, perhaps including a signal peptide at the amino terminus [39]. Sato et al. reported *FGF4* was related to the regulation of bovine embryo development [38]. Consistent with these findings, Feldman et al. also found *FGF4* was associated with trophoblast proliferation in mice, and *FGF4* null mice showed a peri-implantation lethal phenotype [40]. This evidence supported *FGF4* was an important indicator in the growth and development of animals.

*Angiopoietin-like 4* (*ANGPTL4*), known as a novel peroxisome proliferator-activated receptor target gene, is a key regulator of triglyceride, non-esterified fatty acid (NEFA) concentrations, and plasma cholesterol [41]. Just as in the mouse study, *ANGPTL4* is also widely expressed in many bovine tissues, such as liver, subcutaneous adipose tissue, rumen, omasum, abomasum, etc., among which the first two are important tissues for *ANGPTL4* synthesis [42]. Under fasting conditions, its expression level is strongly up-regulated in the liver and adipose tissue [43], which plays an important role in lipid metabolism via inhibition of the lipoprotein lipase (LPL) and stimulated lipolysis [44]. *ANGPTL4* has been recognized as an adipokine in bovine adipose tissue and its expression could affect bovine body fat [45]. Notably, intramuscular fat becomes an important component of maturing muscles, and the mass of skeletal muscle accounts for 40% of total body weight in animals [32], thus it could be speculated that *ANGPTL4* might regulate BW trait by influencing the formation of adipose tissue. Additionally, previous studies have reported *ANGPTL2*, a homologous family gene of *ANGPTL4*, influences the development of the bovine BW trait [24]. Taken together, *ANGPTL4* could be recognized as the candidate gene regulating the BW trait for further research.

*Phospholipase A2* (*PLA2*) is classified into three groups according to their chemical properties and molecular structure, namely cytosolic (*cPLA2*), secretory (*sPLA2*), and Ca^2+^-independent PLA2s [46]. Previous researchers found that activated *cPLA2* could stimulate the release of arachidonic acid [46], which directly suppressed the growth and development of tumor cells [47]. Phosphorylation of *cPLA2* induced by Temozolomide could also cause the suppression of cell growth [48]. More importantly, *cPLA2* is an important regulator in various muscle development. Gluck et al. proposed *cPLA2* activation was essential for the proliferation of bovine aortic smooth muscle cells [49]. In the work by Hirabayashi et al., *cPLA2* alpha was identified to be responsible for striated muscle growth and fertility in mice [50]. In the present study, *cPLA2*, known as *PLA2G4A*, was significantly enriched in two signal transduction pathways, including the MAPK signaling pathway (bta04010) and Ras signaling pathway (bta04014). As mentioned above, the MAPK signaling pathway could stimulate the growth of skeletal muscle [33]. Hence, *cPLA2* was forecasted to be the promising gene affecting the bovine BW trait via the involvement in muscle development.

Integrins are a family of heterodimeric cell-surface adhesion receptors that affect cell-matrix interaction. Some integrins encoding genes, including *integrin alpha-2* (*ITGA2*) and *integrin alpha-11* (*ITGA11*), have been proven to regulate the BW trait of swine and sheep, respectively [51,52]. In this study, *integrin alpha-5* (*ITGA5*) was speculated to be associated with bovine BW trait. *ITGA5* participated in various cellular processes, such as cell adhesion, survival, proliferation, differentiation, and migration of myoblasts [53], adipocytes [54], and cardiac neural crest [55]. Its differential expression was correlated with the organ specificity of tumor metastasis [56], thus *ITGA5* was recognized as a potential biomarker for cancer treatment. Previous studies have shown *ITGA5* could promote the proliferation, migration, and invasion of oral squamous cell carcinoma through activating the PI3K/AKT signaling pathway [57]. Chen et al. demonstrated that *ITGA5* was a mediator for the proliferation and migration of retinal pigment epithelial cells [58]. *ITGA5* knockdown or overexpression could inhibit or accelerate cell growth, respectively. Fang et al. reported *ITGA5* participated in integrin β1 overexpression, which caused growth arrest of breast cancer cell [59]. Larzabal et al. revealed that suppressed *ITGA5* could reduce adherence capacity to fibronectin and inhibit tumor growth in lung cancer cells [60]. In addition to cell growth, *ITGA5* has been proposed to regulate porcine drip loss by mediating cell adhesion and extracellular matrix [61]. However, at present, there is no supporting evidence for *ITGA5* on the weight of beef cattle, thus *ITGA5* as a molecular marker for bovine growth traits needs further investigation.

Combined with previous studies and gene function analysis, except for candidate genes listed above, several known or potential candidate genes were also identified to affect bovine BW trait in this study. *TBC1 domain family member 5* (*TBC1D5*), encoding GTPase-activating protein (GAP) for Rab7, is a high-affinity ligand of the retromer cargo selective complex VPS26/VPS29/VPS35. Previous studies showed *TBC1D5* was an important novel regulator that rerouted ATG9-containing vesicular carriers toward sites of autophagosome formation [62]. Bärlocher et al. illustrated *TBC1D5* could promote the intracellular growth of L. pneumophila [63]. Notably, Zhuang et al. reported *TBC1D5* might play a special role in BW at 18 months of age in Chinese Simmental beef cattle [11]. Consistent with these findings, *TBC1D5* was also identified as the functional gene for the bovine BW trait in the present study. However, the molecular mechanism by which *TBC1D5* influence**s** bovine BW remain**s** to be elucidated.

Previous studies have demonstrated that *SHISA9* affected the growth and development traits such as pre-weaning gain in sheep and BW in beef cattle [24,64]. *Protein shisa-2 homolog 2* (*SHISA2*) identified in this study belongs to the same family as *SHISA9*, which encodes an endoplasmic reticulum protein against both Wnt and FGF signaling to affect the development of Chicken and Xenopus embryos [65,66]. Liu et al. reported *SHISA2* not only regulated F-actin distribution but also directly mediated the maturation of membrane protein for myoblast fusion. Its overexpression could inhibit myoblasts’ proliferation but promote premature fusion [67]. Human *SHISA2* overexpression led to increased cell growth and invasion [68]. In the work by Hu et al., *SHISA2* was identified to be involved in growth and development in duck skeletal muscle [69]. As mentioned previously, skeletal muscle mass accounts for 40% of total body weight in animals [31], thus implying *SHISA2* might regulate BW trait via affecting the development of bovine skeletal muscle.

*Phosphodiesterase* (*PDE1C*) regulates the stability of growth factor receptors such as PDGFRβ [70]. which is highly expressed in the human heart, cardiac myocytes, and mouse heart, but rarely expressed in mouse cardiac myocytes [71,72]. Cai et al. demonstrated that *PDE1C* positively regulated smooth muscle cells (SMCs) growth, proliferation, migration, and neointimal hyperplasia [73]. In agreement, the high expression of *PDE1C* was screened in proliferating human arterial SMCs in primary culture, but not in the quiescent SMCs [74], which indicated this gene was an indicator of cell proliferation. Notably, the previous study by Duan et al. found *PDE1C* might be the candidate gene affecting bovine BW trait through signal-trait GWAS [24]. However, many studies of *PDE1C* mainly rely on human and mouse SMCs, with less research on beef cattle, thus the functions of *PDE1C* in beef cattle should be further investigated.

*Cytochrome c oxidase subunit 6C* (*COX6C*) is eventually transported to mitochondria to form the cytochromec oxidase (COX) complex [75]. Duggan et al. suggested that COX participated in the remodeling of skeletal muscle [76]. The expression levels of COX subunits are different in vertebrate muscle [77]. *COX6C* overexpression could induce cell growth retardation [78]. Therefore, it was speculated that *COX6C* might be a valuable gene for bovine growth and development.

## 5. Conclusions

In conclusion, GWAS-RRM has been recognized as the main analysis model for longitudinal traits as it could decrease FPR and increase statistical powers. Based on this method, the present study mainly revealed four most promising candidate genes (*FGF4*, *ANGPTL4*, *PLA2G4A*, and *ITGA5*) and two significantly enriched pathways regulated bovine BW trait by affecting the growth and development of skeletal muscle and adipose tissue. The function of these candidate genes and pathways have been analyzed and discussed in detail. Moreover, further studies will be necessary to clarify their molecular mechanisms and physiological implications in regulating BW trait. This study not only offers molecular information for genomic selection of bovine growth and development traits but also provides the reference for the large-scale application of GWAS-RRM analysis of longitudinal traits in other livestock and poultry.

## Figures and Tables

**Table 1:** The detected candidate genes affecting body weight trait.

BTA ^1^	SNP	Position ^2^ (bp)	Distance ^3^ (bp)	Gene	*p*-Value ^4^
1	BovineHD0100045595	156,093,740	within	*TBC1D5*	4.60 × 10^−6^
	BovineHD0100003348	10,460,874	243,218	*MRPL39*	2.17 × 10^−6^
	BovineHD0100004041	13,081,890	1,709,537	*NCAM2*	3.07 × 10^−6^
	BovineHD0100046569	114,775,186	56,124	*YWHAH*	3.97 × 10^−6^
	BovineHD0100022686	78,806,582	77,944	*TPRG1*	4.23 × 10^−6^
2	BovineHD0200031068	107,968,173	within	*PTPRN*	3.17 × 10^−7^
	BovineHD0200018897	65,376,344	98,097	/	2.91 × 10^−6^
	BovineHD0200037229	128,220,525	within	*LOC282685*	4.06 × 10^−6^
3	BovineHD0300009402	29,779,589	within	*PHTF1*	3.12 × 10^−6^
4	Hapmap36353-SCAFFOLD29708_3468	64,923,141	62,596	*PDE1C*	2.41 × 10^−6^
	BovineHD0400005814	19,409,767	41,461	*THSD7A*	4.25 × 10^−6^
5	BovineHD0500007511	25,778,691	within	*ITGA5*	1.48 × 10^−6^
	ARS-BFGL-NGS-119234	56,876,453	1314	*SDR9C7*	4.86 × 10^−6^
6	BovineHD0600001415	6,027,009	7189	*BT.87489*	4.30 × 10^−6^
7	BovineHD0700024228	82,801,757	within	*RARS*	1.31 × 10^−6^
	BovineHD0700012290	42,156,049	/	/	4.15 × 10^−6^
8	BovineHD0800029069	98,418,906	within	*ZNF462*	4.06 × 10^−6^
	BovineHD0800009085	29,939,592	90,148	*NFIB*	4.35 × 10^−6^
9	BovineHD0900003150	12,246,543	314,022	RIMS1	2.38 × 10^−6^
10	BovineHD1000029459	101,577,026	within	*TTC8*	7.55 × 10^−8^
11	BovineHD1100004962	15,555,441	within	*LTBP1*	2.54 × 10^−6^
	BovineHD1100011885	40,440,551	109,804	*VRK2*	3.42 × 10^−6^
	BovineHD1100030552	105,125,657	15,309	/	4.45 × 10^−6^
	BovineHD1100012203	41,709,686	965,290	*FANCL*	4.77 × 10^−6^
12	BovineHD1200027798	33,568,673	9620	*SHISA2*	3.40 × 10^−6^
	BovineHD1200026844	26,501,234	386,041	*CHMP3*	4.49 × 10^−6^
	ARS-BFGL-NGS-37745	77,271,637	within	*TMTC4*	4.40 × 10^−6^
13	BovineHD1300017420	60,743,532	within	*ANGPTL4*	4.72 × 10^−6^
14	BovineHD1400018666	66,735,095	95,093	*COX6C*	8.66 × 10^−7^
	BovineHD1400015595	55,998,180	654,428	*KCNV1*	8.50 × 10^−7^
16	BovineHD1600019714	69,438,282	38,338	*PLA2G4A*	3.78 × 10^−6^
	ARS-BFGL-NGS-56551	52,584,126	within	*INTS11*	1.65 × 10^−6^
19	BovineHD1900013251	47,547,492	11,129	*TLK2*	1.39 × 10^−6^
21	BovineHD2100021363	52,053,470	24,294	*LRFN5*	3.23 × 10^−6^
25	BovineHD2500007568	27,048,437	325	*FBRS*	2.45 × 10^−6^
29	BovineHD2900014092	47,651,695	4899	*FGF4*	4.09 × 10^−6^
	BovineHD2900008350	28,354,480	3970	/	2.16 × 10^−6^

^1^ BTA, *Bos taurus* autosome; ^2^ Position on reference genome *Bos_taurus* UMD 3.1; ^3^ Distance between SNP and the nearest gene; ^4^ *p*-values calculated based on Equation (8).

**Table 2:** The most important candidate genes affecting body weight trait.

SNP	BTA ^1^	Position ^2^ (bp)	Distance ^3^ (bp)	Gene	*p*-Value ^4^
BovineHD2900014092	29	47,651,695	4,899	*FGF4*	4.09 × 10^−6^
BovineHD1300017420	13	60,743,532	within	*ANGPTL4*	4.72 × 10^−6^
BovineHD1600019714	16	69,438,282	38,338	*PLA2G4A*	3.78 × 10^−6^
BovineHD0500007511	5	25,778,691	within	*ITGA5*	1.48 × 10^−6^
BovineHD0100045595	1	156,093,740	within	*TBC1D5*	4.60 × 10^−6^
BovineHD1200027798	12	33,568,673	9620	*SHISA2*	3.40 × 10^−6^
Hapmap36353-SCAFFOLD29708_3468	4	64,923,141	62,596	*PDE1C*	2.41 × 10^−6^
BovineHD1400018666	14	66,735,095	95,093	*COX6C*	8.66 × 10^−7^

^1^ BTA, *Bos taurus* autosome; ^2^ Position on reference genome *Bos_taurus* UMD 3.1; ^3^ Distance between SNP and the nearest gene; ^4^ *p*-values calculated based on Equation (8).

## Data Availability

The raw data underlying our findings has been submitted to Dryad Digital Repository, which is available from the following link: https://doi.org/10.5061/dryad.4qc06, accessed on 1 July 2021.

## Supplementary Materials

The following are available at supplementary materials: https://www.mdpi.com/article/10.3390/ani11092524/s1, Table S1: The descriptive statistics of bovine body weight at different months of age; Table S2: The information of significant SNPs and candidate genes affecting BW detected by GWAS-RRM; Table S3: KEGG pathways significantly enriched with candidate genes affecting BW trait; Table S4: The candidate genes affecting BW trait reported in the major domestic animals QTL database.
